# High-quality reference genome of cowpea beetle *Callosobruchus maculatus*

**DOI:** 10.1038/s41597-024-03638-w

**Published:** 2024-07-18

**Authors:** Hao-Ran Lu, Chu-Yang Mao, Li-Jie Zhang, Jin-Wu He, Xie-Shuang Wang, Xin-Ying Zhang, Wei-Li Fan, Zheng-Zhong Huang, Le Zong, Chu-Han Cui, Feng-Ming Wu, Xue-Li Wang, Zhen Zou, Xue-Yan Li, Si-Qin Ge

**Affiliations:** 1 State Key Laboratory of Integrated Management of Pest Insects and Rodents, Institute of Zoology, Chinese Academy of Sciences, Beijing, China; 2 University of Chinese Academy of Sciences, Beijing, China; 3 Key Laboratory of Genetic Evolution & Animal Models, Kunming Institute of Zoology, Chinese Academy of Sciences, Kunming, Yunnan China; 4 Science and Technical Research Center of China Customs, Beijing, China; 5 Northwestern Polytechnical University, Xian, China; 6 State Key Laboratory of Zoological Systematics and Evolution, Institute of Zoology, Chinese Academy of Sciences, Beijing, China; 7 College of Life Sciences, Hebei University, Baoding, China; 8 Yunnan Key Laboratory of Biodiversity Information, Yunnan, 650223 China

**Keywords:** Developmental biology, Genetics

## Abstract

*Callosobruchus maculatus* is one of the most competitive stored grain pests, which causes a great loss to agricultural economy. However, due to an inadequacy of high-quality reference genome, the molecular mechanisms for olfactory and hypoxic adaptations to stored environments are unknown and require to be revealed urgently, which will contribute to the detection and prevention of the invasive pests *C. maculatus*. Here, we presented a high-quality chromosome-level genome of *C. maculatus* based on Illumina, Nanopore and Hi-C sequencing data. The total size was 1.2 Gb, and 65.17% (797.47 Mb) of it was identified to be repeat sequences. Among assembled chromosomes, chromosome 10 was considered the X chromosome according to the evidence of reads coverage and homologous genes among species. The current version of high-quality genome provides preferable data resources for the adaptive evolution research of *C. maculatus*.

## Background & Summary

*Callosobruchus maculatus* (Coleoptera: Chrysomelidae), commonly known as cowpea weevil, is a kind of universal stored grain insect pest^1^. *C. maculatus* feeds on a diverse range of legume seeds, and was originally distributed in the tropical and subtropical areas especially Africa and South Asia^2^. However, with the global climate change and international communication, *C. maculatus* were observed in wider regions^3^ and caused great losses to agricultural grain storage. Each female beetle lays huge amount of eggs on the surface of seeds, then the first instar larvae hatch and tunnel into the seeds where larvae will grow up and pupate by feeding on cotyledon, and complete its lifecycle by emerging as adult beetle^1^. To control pests and reduce food waste, dozens of approaches have been implemented in the past decades^4–8^.

**Fig. 1:**
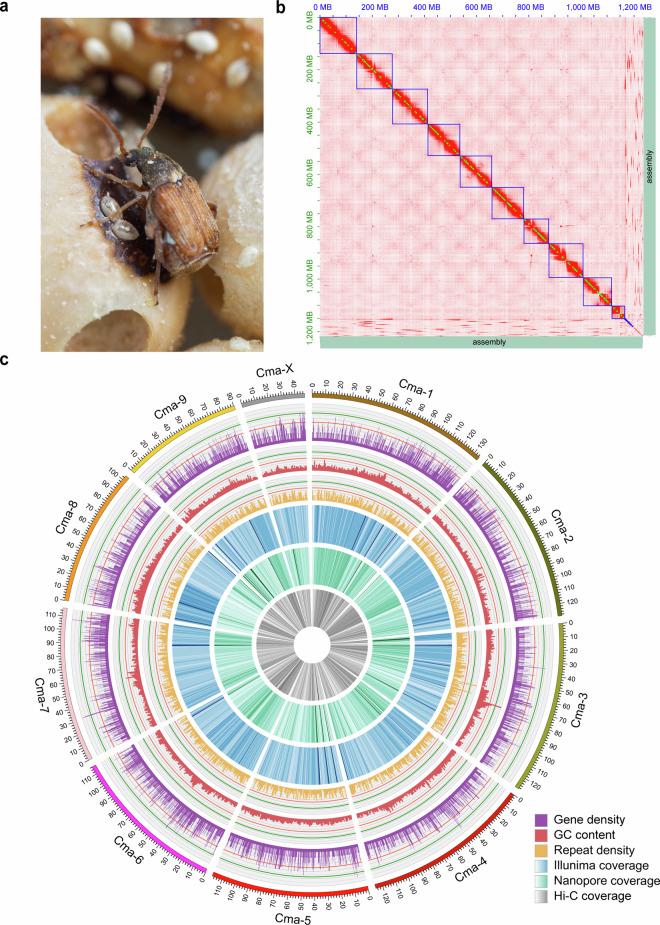
Photograph of *C. maculatus* and Hi-C interaction heatmap. **(a)** A picture of male *C. maculatus* on cowpea. **(b)** Hi-C heatmap of *C. maculatus* genome showing interactions among the 10 assembled chromosomes at 1 Mb resolution. Boxes in blue and green colour indicate scaffolds and contigs. **(c)** The relative gene density, GC content, repeat density, as well as the coverage of second-generation, third-generation, and Hi-C sequencing data in chromosome level are represented from the outside to the center of the circle.

A high-quality chromosome-level genome for *C. maculatus* (Fig. 1a) supplies a practical and much-needed resource for *C. maculatus* agricultural pest control due to its immeasurably damage to stored products, which is also valuable for understanding the molecular mechanisms of its physiological activity and evolution relationships in Coleoptera. In the past, a contig-level genome of *C. maculatus* had been assembled^9^, but it was smaller than the expected genome size due to unknow reasons^10^. The *C. maculatus* genome is highly heterozygous with large proportion of repetitive DNA sequences, rendering substantial challenges for the genome assembly which is also a momentous reason for difference between *K*-mer analysis and flow cytometry^11,12^ in genome size^13^. Compared to the result of *K*-mer analysis, flow cytometry assay experiment was more credible^11,12^. The *K*-mer distribution analysis of the new assembled genome also indicated *C. maculatus* genome was large with high heterozygosity compared to other known Coleoptera species^14,15^. To address the challenges posed by high heterozygosity and repetitive DNA sequences, long-read sequencing (Nanopore), next-generation sequencing (Illumina), and high-throughput chromosome conformation capture (Hi-C) mapping have been employed, and these approaches have proven successful in achieving a high level of completeness and continuity in genome assemblies for various plant species^16,17^ as well as animals^14,18^.

To facilitate *C. maculatus* control and prevention, a 1.2 Gb novel high-quality chromosome-level genome of *C. maculatus* was provided in this research stemming from the new whole genome assembly and correct strategies. Here, we sequenced and assembled the high-quality chromosomal-level reference genome of cowpea weevil combining Illumina, Nanopore and Hi-C sequencing technologies. The current version of high-quality genome offers superior data resources study of adaptive evolution in of *C. maculatus*.

## Methods

### Sampling and genome sequencing

Adult insects of *C. maculatus* were intercepted in imported cowpeas originally from Nigeria and transferred via Ethiopia by Beijing Customs, P.R. China, and then inbreed for about 20 generations with full sib-pair mating strategy which were reared on 28 ± 2 °C, 75% relative humidity, 16 h/8 h light/dark photoperiod in ΦA = 90 mm petri dishes and nurtured with cowpeas. Genomic DNA were extracted from 10 male beetles for sequencing using DNeasy Blood & Tissue Kit (QIAGEN, Germany).

Short reads libraries (insert size: 350 bp) were generated using a Next Ultra DNA Library Prep Kit (NEB, USA), and sequenced on Illumina Novaseq6000 platform (PE-150) at Novogene. The raw reads ran through quality control before the next analysis. For short-read data (i.e., Illumina data), the reads which included adapters, the low-quality reads and N bases were removed, a total of 142.48 Gb clean data was obtained (Table 1). The rest of short data were used to estimate genome size based on the *K*-mer size using Jellyfish^19^ (2.3.0) with option -s 1.2 G. The genome size was also estimated by flow cytometry (Table 2) using 5 males and 5 female beetles as described before^18^. Additionally, these short-read data were used to correct the potential base errors in de novo genome assembly. For long-read data (i.e., Nanopore data), genomic DNA was extracted to construct 20-kb libraries and used for Oxford Nanopore sequencing platform at NextOmics and generated 137.33 Gb raw data (Table 1).

**Table 1:** Summary of sequence reads.

Method	Plarform	Data (Gb)
Short reads	Illumina Novaseq6000	142.48
Long reads	Nanopore PromethION	137.33
Hi-C reads	Illumina Novaseq6000	668.22

**Table 2:** Result of flow cytometry assay.

Number	C-value (pg)		C-value (Gb)
	*Callosobruchus maculatus*	*Drosophila melanogaster*	*Callosobruchus maculatus*
1	889.00	136.06	1.18
2	917.00	142.00	1.16
3	874.00	132.49	1.19
4	887.00	133.00	1.20
5	890.00	135.28	1.18
Average	891.40	135.77	1.18

The Hi-C sequencing was also conducted at NextOmics, which followed the standard protocol and was sequenced on the Illumina NovaSeq6000 platform. Restriction enzyme *DpnII* was used to lyse and digest the isolate cells which were from sliced tissues and cross-linked before overnight. The cohesive ends were blunted, reversed, and marked with biotin-14-dATP and purified the DNA by removing biotin from unligated ends. DNA was sheared to 200–300 bp fragments via a Covaris M220 and pulled down the point ligation junctions by Dynabeads MyOneTM Streptavidin C1 after size selection with AMPure XP beads. Finally, a total of 668.22 Gb raw data of Hi-C sequencing was obtained and used to assist genome assemble on chromosome-level.

For transcriptome sequencing of adult female/male *C. maculatus*, each sample consisted of three beetles, with three replicates for each stage or gender. RNA was extracted using Trizol (Invitrogen, USA), library construction, sequencing on the Illumina NovaSeq6000 platform (PE-150) and quality control was performed in Novogene, and clean data was generated for further analysis.

### *De novo* genome assembly

To combine the advantages of high accuracy of second-generation sequencing and long reads of third-generation sequencing^20^, this research employed the strategy that both sequencing methods were combinedly used for genome assembly. Genomic DNA was extracted and sequenced via Illumina platform and in prior to the Oxford Nanopore sequencing, the *K*-mer distribution analysis indicated that the genome size of *C. maculatus* was 1,358.89 Mb based on 21-mers using the illumina data, which was used to evaluate its size and characteristics, and consistent with the result of flow cytometry assay experiment (1,182.11 Mb, Table 2).

The long-read data was used to assemble the primary genome. NextDenovo^21^ (2.3.1) package was used to assemble the genome, which had several scripts and seq_stat, a binary script to statistic the information of sequencing data, is one of them. After using seq_stat with option -g = 1,200,000,000 to generate the seed cutoff value, which was required by the main program, run command NextDenovo, the main program, to get the primary genome with default configure. The primary genome was adjusted with option -a = 50 by purge_dups^22^ from 3 Gb into 1.2 Gb which was confirmed by *K*-mer analysis and flow cytometry previously^23^. NextPolish^24^ (1.2.3) package was used to polish the primary genome with default configure, which integrated the short-read data and the assembled the 1.2 Gb draft genome with an N50 of 1.03 Mb.

The Hi-C paired-end reads were mapped to the above draft genome iteratively using chromap^25^ (0.2.3) and yahs^26^ (1.2a1). Finally, juicebox^27,28^ (2.18) was applied to correct the contig orientation and move the suspicious fragments into unanchored scaffolds via visual exploration of Hi-C heatmaps. After all, 10 assembled chromosomes (Fig. 1b and Table 3), which holds 9 autosomes and one X chromosome (2n = 20, n = 9 + X), consistent with that by karyotype analyses^23,29^. Totally, 78.5% fragments were anchored to the assembled chromosomes, and the genome length is 1,222.50 Mb (N50 = 117.71 Mb) (Fig. 1b and c).

**Table 3:** Statistics of *C. maculatus* genome.

Statistics	Previous version^10^	This version
Genome size (Gb)	1.01	1.22
Total number	15,778	488
N50 length (Mb)	0.15	117.71
Max length (Mb)	2.10	134.95
GC content (%)	37.52	37.58
BUSCO (completeness, %)	89.25	98.39
Illumina reads mapping rate (%)	—	98.94
Nanopore reads mapping rate (%)	—	99.65

### Identification of X chromosomes

**Fig. 2:**
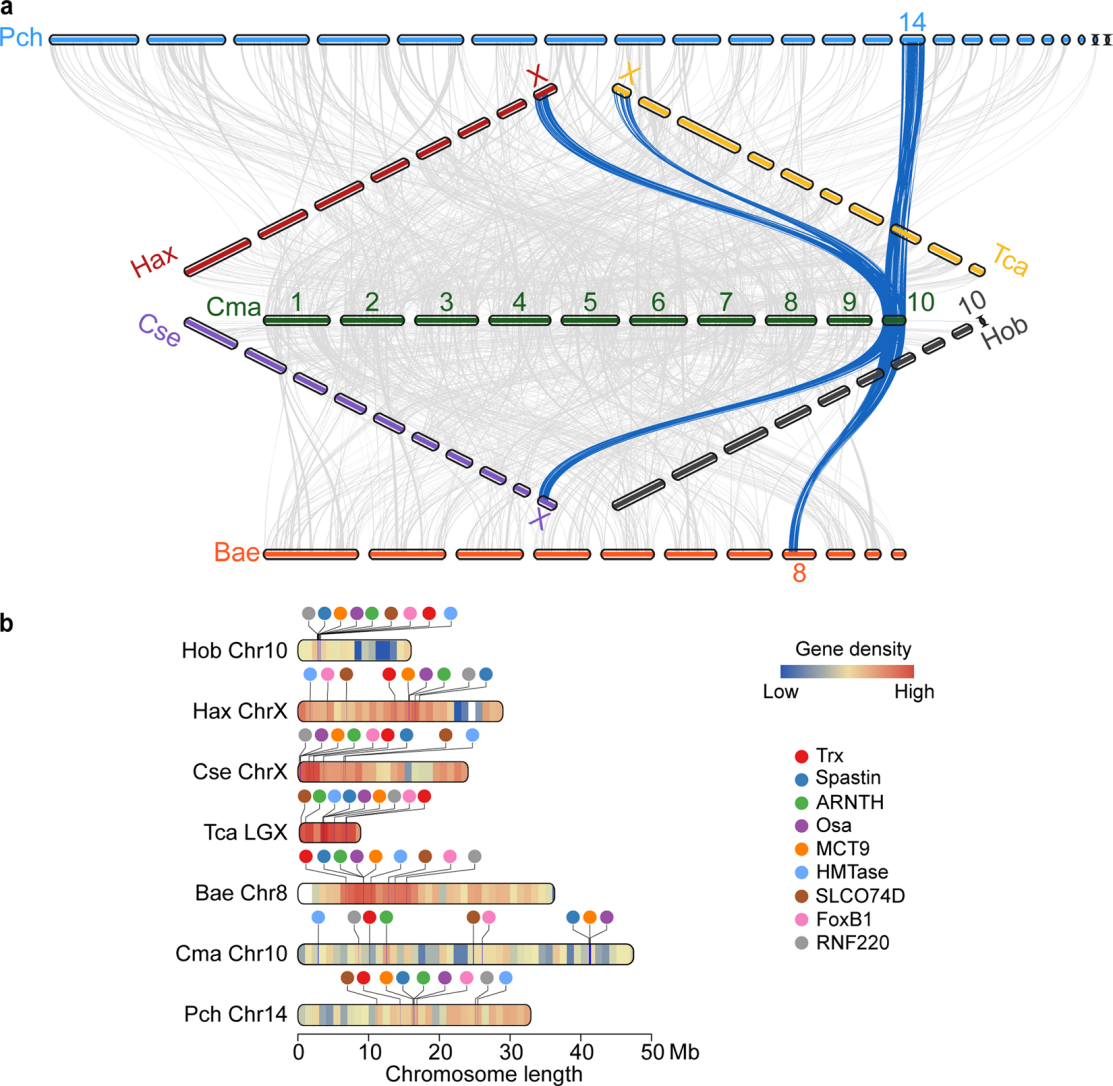
Chromosomes synteny and comparative analysis on chromosome X. **(a)** Gene synteny analysis of seven coleopteran species. Homologous genes are linked by grey lines between chromosomes, while X-chromosome homologous genes are linked by blue lines. **(b)** Gene density of seven coleopteran species X-chromosomes (including predicted chromosomes) is showed from blue (low) to red (high), and white blank meant that no gene are annotated. Nine key genes are located on the chromosomes. Hob: *Holotrichia oblita*, Psh: *Psylliodes chrysocephala*, Bra: *Brassicogethes aeneus*, Cma: *C. maculatus*, Tca: *T. castaneum*, Hax: *H. axyridis*, Cse: *C. septempunctata*.

Female and male beetle transcriptomes proved that Chr-10 is X-chromosome since female beetle transcripts showed significant larger coverage on Chr-10 (Table 4). In addition, we also performed integrating collinearity analysis of multiple homologous species genomes to identify X chromosomes. Chromosome X (Chr-X) is a distinctive group, which had been identified in *Tribolium castaneum* (GCA_000002335.3)^30^, *Harmonia axyridis* (GCA_914767665.1)^31^, and *Coccinella septempunctata* (GCA_907165205.1)^32^, and analyzed among the seven Coleoptera species (Fig. 2a). Furthermore, it was reported that several genes, including *Trx*, *Spastin*, *ARNTH*, etc., were located on the Chr-X in *T. castaneum*, which could be found in all other six Coleoptera species Chr-X (Fig. 2b). Besides, the GO enrichment analysis of all seven Chr-X showed a strong connection to sexual reproduction (Supplementary Fig. 1).

**Table 4:** Coverage between female and male beetles on chromosomes.

Chromosome	Ratio[log10(F/M)]
Chr 1	0.02760
Chr 2	0.02135
Chr 3	0.02817
Chr 4	0.00607
Chr 5	0.01941
Chr 6	0.02911
Chr 7	0.01799
Chr 8	0.01830
Chr 9	0.02676
Chr 10	0.24868

### Repeat annotation

**Fig. 3:**
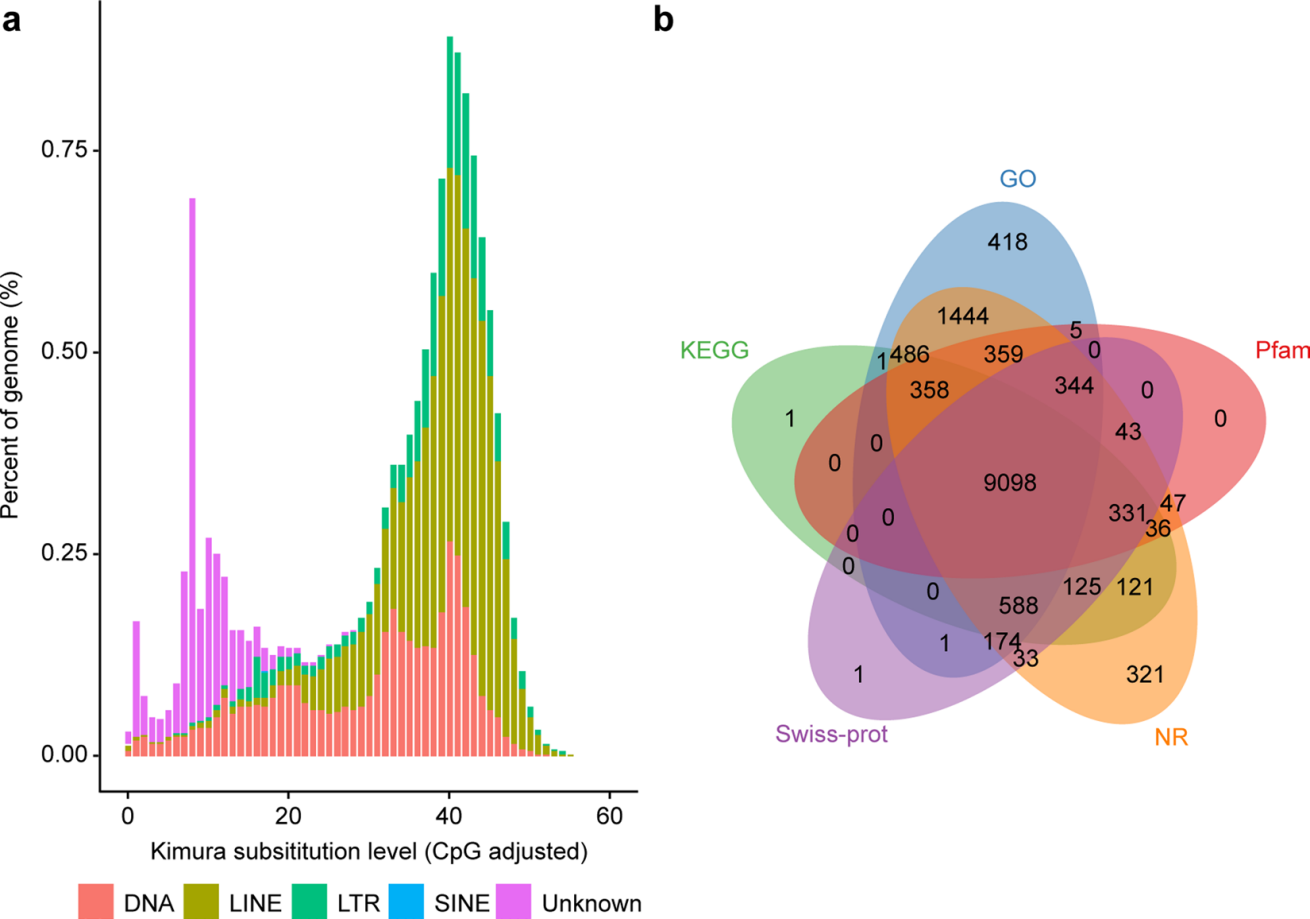
Genome functional annotation of whole genome. **(a)** Taking Repbase as the library, the tandem repeat sequences (TEs) divergence distribution map was obtained by RepeatMasker annotation. The abscissa shows the divergence between the TEs annotated in the *C. maculatus* genome and the corresponding sequences in Repbase; the ordinate is the percentage of TEs in the genome under a specific divergence, and different TEs are marked with different colours. **(b)** Venn diagram how the overlap of five databases, Pfam, Swiss-port, KEGG, GO and NR, used in the annotation. 14,458 genes are annotated from five databases and 9,098 genes are in all the databases.

Repetitive sequence annotation was divided into two types: homologous sequence alignment and *ab initio* prediction. The homologous sequence alignment was based on the repeat sequence database (RepBase library version: RepeatmaskerEdition-20181026), using Repeatmasker^33^ (4.1.5) and RepeatProteinMasker to identify the repetitive sequence had known. *Ab initio* prediction used LTR FINDER^34^ (0.3.1), RepeatScout^35^ (1.0.6) and RepeatModeler^36^ (2.0.4) combined with Repbase nucleotides library and Repbase proteins library. In the beginning, *de novo* repeat library was established, and then used Repeatmasker to predict them. In addition, in the method of *ab initio* prediction, tandem repeat finder (TRF)^37^ (4.0.9) was applied to find tandem repeats (TEs) in the draft genome. Integrated with the result of *ab initio* prediction, 797.47 Mb (65.17%) repeat elements were identified totally (Fig. 3a).

### Gene structure prediction

The strategy of Gene structure prediction combined multiple prediction methods including homology prediction (seven species), *ab initio* prediction and RNA sequences-based prediction. Homology prediction was to compare the coding protein sequence of *Drosophila melanogaster* (GCA_000001215.4)^38^, *Diabrotica virgifera* (GCA_917563875.2)^39^, *T. castaneum*, *Anoplophora glabripennis* (GCA_000390285.2)^40^, *Sitophilus oryzae* (GCA_002938485.2)^41^, *Leptinotarsa decemlineata* (GCA_000500325.2)^42^, and *Aethina tumida* (GCA_024364675.1)^43^ with the genome sequence of *C. maculatus* via blast and genewise to predict the gene structure in the genome. Geneid^44^ (1.4.5), Augustus^45^ (3.5.0), GlimmerHMM^46^ (3.0.4), SNAP^47^ (2006-07-28), and Genscan^48^ (1.0) were employed with default configure in the *ab initio* prediction which relied on the statistical characteristics of genome sequence data, codon frequency and exon-intron distribution, to predict gene structure. Program to Assemble Spliced Alignments (PASA)^49^ (2.5.3) and Cufflinks^50^ (2.2.1) were applied in the RNA sequences-based prediction method with default settings. Based on the above prediction results, combined with the transcriptome comparison data, the EVidenceModeler (EVM)^49^ (2.1.0) and Liftoff^51^ (1.6.3) was used to integrate the gene sets predicted by various methods into a non-redundant and more complete gene set with different weights (Supplementary Fig. 2). Finally, used PASA to correct the EVM annotation results combined with the transcriptome assembly results, added untranslated region (UTR) and variable splicing and other information to get the final gene set. Using homology prediction, *ab initio* prediction and RNA sequences-based prediction, totally 14,458 genes were predicted (Table 5, Fig. 3b), and 91.9% proteins were conserved in BUSCO^52^ (5.4.1) analysis with protein mode based on Insecta odb10 (Table 6). And using the same method for previous version of the genome showed that annotated gene-sets have completeness value was 84.2% with 63.6% single copy, while the original completeness value was 75% which based on arthropod datasets^10^.

**Table 5:** Comparison of gene structures in related species.

Species	Number	Average transcript length (bp)	Average CDS length (bp)	Average exons per gene	Average exon length (bp)	Average intron length (bp)
*Agl*	14,815	16,288.47	1,520.12	5.59	271.72	3,214.43
*Atu*	14,076	6,198.17	1,474.90	5.93	248.78	958.34
*Cma*	14,458	34,576.66	1,481.93	6.31	234.84	6,232.20
*Dvi*	20,592	37,550.53	1,232.27	4.51	273.25	10,348.22
*Lde*	14,000	13,981.38	1,386.78	5.06	273.80	3,098.36
*Sor*	15,044	22,712.70	1,561.81	6.35	246.01	3,954.54
*Tca*	12,863	6,802.05	1,565.09	5.30	295.38	1,218.29

*Agl, Atu, Cma Dvi, Lde, Sor* and *Tca* represent *A. glabripennis, A. tumida, C. maculatus, D. virgifera, L. decemlineata, S. oryzae* and *T. castaneum*.

**Table 6:** BUCSO evaluation for genome assembly.

	Previous version^10^		New version		
	genome assembly	protein annotation	Hi-C genome	polish genome	protein annotation
Complete BUSCOs	89.2%	84.2%	98.4%	98.1%	91.8%
Complete and single copy BUSCOs	84.2%	63.6%	85.8%	84.4%	82.7%
Complete and duplicated BUSCOs	5.0%	20.6%	12.6%	13.7%	9.1%
Fragmented BUSCOs	3.2%	5.3%	0.3%	0.5%	2.3%
Missing BUSCOs	7.6%	10.5%	1.3%	1.4%	5.9%

### Gene function prediction and Non-coding RNA annotation

The gene functions were identified by aligning to Swiss‐prot, the nonredundant sequence databases: Nucleotide collection (NR and NT), eukaryotic orthologous groups of proteins (KOG), KEGG, TrEMBL and using BLAST^53^ (2.15.0 + ) with an E-value cutoff of 1e-5. The Blast2GO^54^ (6.0) was employed to annotate gene functions in the GO database based on the aligned results from the NR database. The molecular pathways of predicted genes, which might be involved, were detected through search and annotation for the KEGG database. Using Interproscan^55^ (5.62–94.0) to search in the Pfam (35.0), PRINTS (42.0), SMART (9.0) databases, known motifs and domains in the *C. maculatus* genome were found. The domain boundaries of interesting proteins were searched on the Pfam website. In all, 14,013 of 14,458 genes were supported by functional annotation from the databases (Fig. 3b). It is worth noting that several genes related to hypoxia, odorant, and immunity were not well annotated in the previous genome, such as olfactory receptors and clip-domain serine proteases which had been spot checked.

According to the structural characteristics, tRNAscan-SE^56^ (1.3.1) was used to identify the tRNA in the genome. Meanwhile, because of the highly conservative of it, the rRNA sequences of related species were used as a reference sequence and aligned with *C. maculatus* genome via blast. Additionally, the covariance model of the Rfam family was used, and the INFERNAL^57^ (1.1.4) that came with Rfam to predict the information of miRNA and snRNA on the genome. There were 493,139 bp non-coding RNA, and most of them is tRNA (2,827 genes for 206,000 bp), while it was 6,948 tRNA genes in the previous genome^10^.

## Data Records

The whole raw data has been deposited at the NCBI Sequence Read Archive under BioProject number PRJNA1048654 and BioSample ID SAMN38657795 for *C. maculatus*. Raw sequencing data (Illumina, Nanopore, Hi-C and RNA-seq data) have been deposited in the Sequence Read Archive database as SRP477247^58^. The final genome assembly and gene annotation results have been deposited in GenBank^59^ and Figshare^60^.

## Technical Validation

To evaluate the completeness of the assembly, BWA^61^ (0.7.17) was used to align the short-reads data with genome while Minimap2^62^ (2.17) aligned the long-reads data and the coverage depth for assembled chromosomes were calculated via SAMtools^63^ (1.16.1) (Table 7). The chromosome-level genome was also evaluated via BUSCO^52^ (5.4.1) which was compared with Insecta odb10 with 1367 genes, and the results showed that 98.4% and 0.3% conserved core genes were identified as completed and fragmented (Table 6). These results showed that the assembled *C. maculatus* chromosome-level genome has an elevated level of completeness.

**Table 7:** Statistics of genome mapping.

	mapped length (bp)	number mapped reads	percent of genome
Short-read sequence	140,893,195,950	985,181,432	98.94%
Long-read sequence	137,175,775,791	53,560,382	99.65%
Hic sequence	655,131,101,700	5,986,042,949	98.56%

### Supplementary information

Supplementary information

## Data Availability

All commands and pipelines utilized in the data processing were executed in accordance with the manuals and protocols of the respective bioinformatics software. In instances where detailed parameters were absent, default parameters were employed. The version of the software used is delineated in the Methods section. Notably, no custom programming or coding was incorporated.
