# Supplementary material for: High-quality reference genome of cowpea beetle Callosobruchus maculatus
Source: Sci Data. 2024 Jul 18;11:799. doi: 10.1038/s41597-024-03638-w (PMC11258224; doi:10.1038/s41597-024-03638-w)
Supplement: Supplementary file 1 — Supplementary information [file 41597_2024_3638_MOESM1_ESM.docx]

**Description of Additional Supplementary Files**

Fig. S1 GO enrichment analysis of X-chromosomes.

Fig. S2 Statistics of gene set evidence supports.


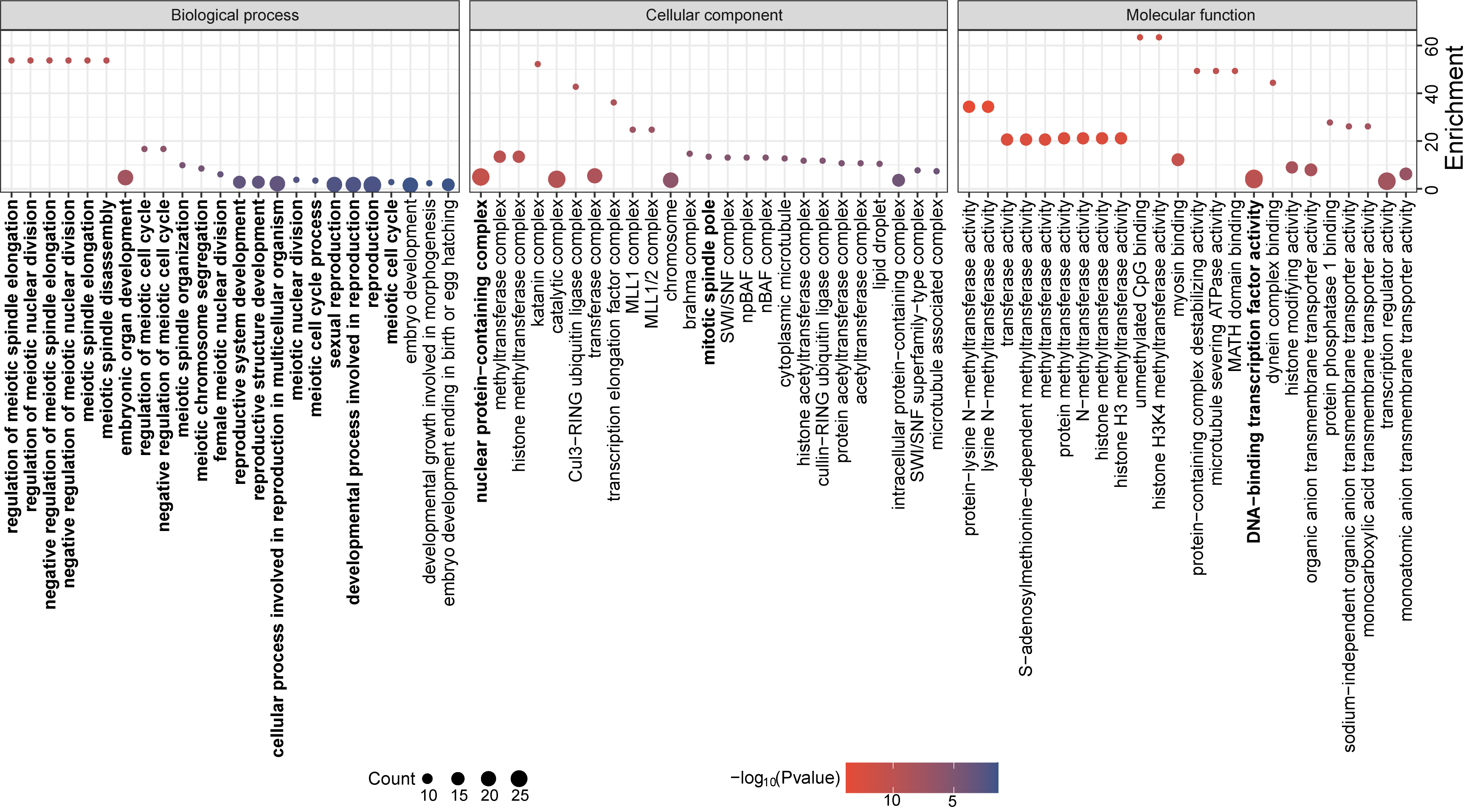


**Fig. S1 GO enrichment analysis of X-chromosomes.**

Top 25 of every GO class were presented and terms related to meiosis and sexual reproduction.


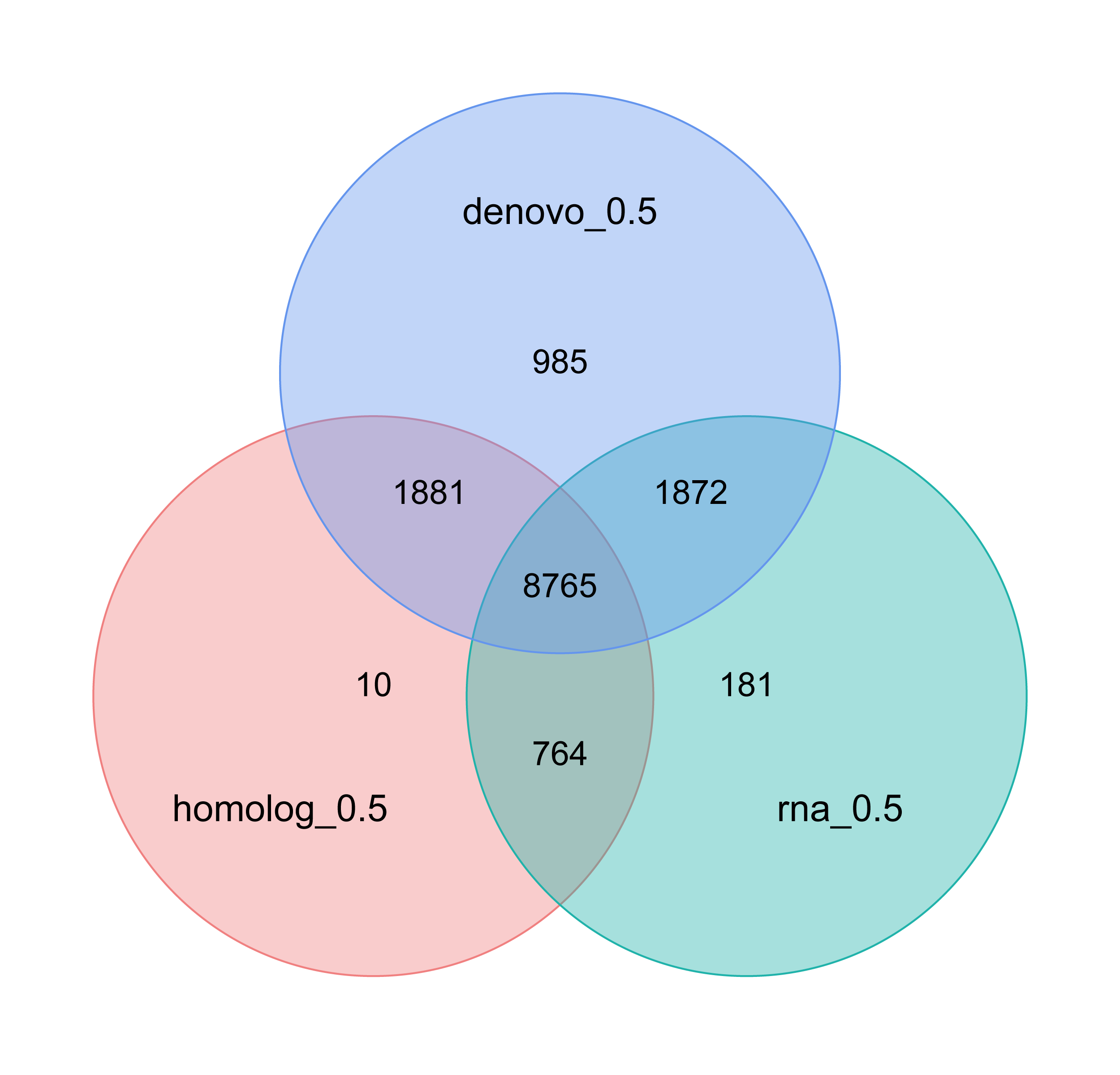


**Fig. S2 Statistics of gene set evidence supports.**

*De novo*, the genes supported by de novo prediction in EVM integration; Homolog, genes supported by homology prediction in EVM integration; RNA, genes supported by RNA-seq in EVM integration; each evidence support is based on gene overlap greater than 50% as the standard. The numbers indicate the number of genes.
